# Metabolomics of Photosynthetically Active Tissues in White Grapes: Effects of Light Microclimate and Stress Mitigation Strategies

**DOI:** 10.3390/metabo11040205

**Published:** 2021-03-30

**Authors:** Andreia Garrido, Jasper Engel, Roland Mumm, Artur Conde, Ana Cunha, Ric C. H. De Vos

**Affiliations:** 1 Centre for the Research and Technology of Agro-Environmental and Biological Sciences (CITAB), University of Minho, Campus de Gualtar, 4710-057 Braga, Portugal; arturconde@bio.uminho.pt; 2 Business Unit Bioscience, Wageningen Plant Research, Wageningen University and Research (Wageningen-UR), P.O. Box 16, 6700 AA Wageningen, The Netherlands; jasper.engel@wur.nl (J.E.); roland.mumm@wur.nl (R.M.); ric.devos@wur.nl (R.C.H.D.V.); 3 Business Unit Biometris, Wageningen Plant Research, Wageningen University and Research (Wageningen-UR), P.O. Box 16, 6700 AA Wageningen, The Netherlands; 4 Centre of Molecular and Environmental Biology (CBMA), University of Minho, Campus de Gualtar, 4710-057 Braga, Portugal; 5 Centre of Biological Engineering (CEB), University of Minho, Campus de Gualtar, 4710-057 Braga, Portugal

**Keywords:** grape berry tissues, light microclimate, irrigation, kaolin, metabolomics, photosynthesis

## Abstract

The effects of climate change are becoming a real concern for the viticulture sector, with impacts on both grapevine physiology and the quality of the fresh berries and wine. Short-term mitigation strategies, like foliar kaolin application and smart irrigation regimes, have been implemented to overcome these problems. We previously showed that these strategies also influence the photosynthetic activity of the berries themselves, specifically in the exocarp and seed. In the present work, we assessed the modulating effects of both canopy-light microclimate, kaolin and irrigation treatments on the metabolic profiles of the exocarp and seed, as well as the potential role of berry photosynthesis herein. Berries from the white variety Alvarinho were collected at two contrasting light microclimate positions within the vine canopy (HL—high light and LL—low light) from both irrigated and kaolin-treated plants, and their respective controls, at three fruit developmental stages (green, *véraison* and mature). Untargeted liquid chromatography mass spectrometry (LCMS) profiling of semi-polar extracts followed by multivariate statistical analysis indicate that both the light microclimate and irrigation influenced the level of a series of phenolic compounds, depending on the ripening stage of the berries. Moreover, untargeted gas chromatography mass spectrometry (GCMS) profiling of polar extracts show that amino acid and sugar levels were influenced mainly by the interaction of irrigation and kaolin treatments. The results reveal that both photosynthetically active berry tissues had a distinct metabolic profile in response to the local light microclimate, which suggests a specific role of photosynthesis in these tissues. A higher light intensity within the canopy mainly increased the supply of carbon precursors to the phenylpropanoid/flavonoid pathway, resulting in increased levels of phenolic compounds in the exocarp, while in seeds, light mostly influenced compounds related to carbon storage and seed development. In addition, our work provides new insights into the influence of abiotic stress mitigation strategies on the composition of exocarps and seeds, which are both important tissues for the quality of grape-derived products.

## 1. Introduction

The grapevine (*Vitis vinifera* L.) is a perennial woody plant cultivated in many regions worldwide, spreading across temperate to semi-dry areas, but mainly in the latitudes spanning from 30 to 50 degrees [1]. Viticulture represents an important agronomic activity with high socioeconomic relevance, due to the great diversity of grape derived-products consumption (e.g., table grapes, raisins and wine) [2], and its by-products (e.g., pomace, skins and seeds) [3]. According to the International Organization of Vine and Wine (OIV), European viticulture accounts for 60% of the world’s wine production; Portugal is the 11th world and 5th European wine producer [4].

The *terroir*, unique to each wine-growing area, compiles a complex and interacting system of factors including specific soil, topography, climate, landscape characteristics and biodiversity features, in combination with applied viticultural practices [5]. Together, these features influence the canopy microclimate, grapevine physiology, and, consequently, the grape berry composition and wine quality [6]. However, the intensification of abiotic stress due to a changing climate is already experienced in the Mediterranean regions, such as extended summer droughts and higher radiation and temperatures [7,8], imposing negative impacts on grapevine phenology, physiology, and productivity, and urges for measures to be taken [9,10].

Both short and long term strategies are being implemented in viticulture to maintain or even improve plant productivity and fruit quality under abiotic stress conditions [11]. Long-term strategies encompass the relocating of vineyards to cooler sites or sites with lower solar exposure, and the selection of appropriate rootstocks and breeding for stress resistant varieties [12]. Short-term measures include some existing viticultural practices, such as canopy management [13], vine shadings [14], the introduction of cover cropping [15], smart irrigation [16], and foliar kaolin applications [17]. Kaolin, Al_2_Si_2_O_5_(OH)_4_, is a white inert clay mineral that reflects solar radiation. Not only does it reflect damaging ultraviolet (UV) and heat-generating infrared radiation (IR), but also photosynthetically active radiation (PAR) in a less significant manner [18]. Previous works demonstrated the beneficial effects of kaolin on both grapevine leaves, like a decrease in leaf temperature at midday with a parallel increase in both photosynthetic efficiency and photoassimilate synthesis [17,19,20], and the biochemical composition of the berries, including an increase in phenylpropanoids and flavonoids [21,22].

Light is a key factor in the physiology of the plant, enabling photosynthesis [18,23] and related processes, including shoot development and biosynthesis of an array of primary and secondary metabolites [24,25,26]. In fact, photosynthesis is not exclusive to leaves, but it can also occur in other green tissues including reproductive organs like fruits and seeds [27]. We previously characterized the photosynthetic activity of the two most photosynthetic competent grapevine fruit tissues, the exocarp (skin) and seed integuments [28], and of berries growing at contrasting light microclimates that naturally occur inside the plant canopy [18,23]. The so-called Low Light (LL) grape berry clusters grow in the shaded inner zones of the canopy, where they are exposed to only diffuse, reflected and transmitted light and lower temperatures (approx. 50 μmol photons m^−2^ s^−1^ and a temperature of 26 °C), while the High Light (HL) clusters are exposed to both more direct or reflected sunlight and higher temperatures for the greatest part of the day (approx. 150 μmol photons m^−2^ s^−1^ and a temperature of 30 °C) [18]. Overall, our previous studies showed that the exocarp and seeds from HL berries exhibited a significantly higher photosynthetic capacity than the LL berries, especially at their green stage [18,23]. Both tissues showed highest maximum quantum efficiency (F_v_/F_m_) and photosynthetic capacity (ETR_m_) at the green stage, with the exocarp extending its activity up to the mature stage while seed photosynthetic activity was more restricted to the green and *véraison* stages [18,23].

Abiotic stress mitigation strategies like irrigation and foliar kaolin application may have implications on the amount and spectrum of light received by both leaves and fruits: foliar kaolin will directly alter the light reflection inside the canopy [29], while irrigation may indirectly lead to more shading due to an enhanced vegetative growth [30]. Previously, we proved that such kaolin treatment also increased the photosynthetic activity of both exocarps and seed integuments of LL berries [18]. Irrigation lowered the photosynthetic activities in seeds of HL berries at both *véraison* and mature ripening stages, especially under kaolin treatment [18].

Each grape berry tissue (exocarp, mesocarp and seed) contains numerous compounds [31] that are important for wine quality [32], such as sugars, organic acids, amino acids, phenolic compounds and conjugated aroma compounds. The exocarp contains most of the flavonoids, which include flavonols such as quercetin glycosides, flavan-3-ols such as catechins, and in red cultivars also anthocyanins such as delphinidin-glycosides [33]. The mesocarp is the primary site for the accumulation of sugars, hydroxycinnamic acids and organic acids [33]. In the seed coats, high levels of tannins (polymers of flavan-3-ols) are observed [34], while the endosperm contains mainly storage lipids [35]. Both the exocarp and seed contain photosynthetic pigments like chlorophylls and carotenoids [18,23].

The photosynthesis of fruits is generally linked to the biosynthesis of both primary and secondary metabolites [36]. For instance, it has been reported for tomatoes that the photosynthesis of the fruit contributes 15–20% of the carbon assimilates needed for fruit growth, although they represent only a minor part compared to the assimilates imported from the leaves [37]. However, Lytovchenko et al. [38] mentioned that tomato fruit photosynthesis is not required for fruit energy metabolism nor for providing assimilates for growth, but is essential for properly timed seed development. In addition, in the grape berry, photosynthesis may contribute to the total carbon balance by supplying about 10% of the carbon needed for fruit development and by recycling about 40% of the carbon lost by mitochondrial respiration [39]. Moreover, transcriptomics [40,41,42], proteomics [43], metabolomics [41,43,44], and integration of omics data into biosynthetic networks [45,46], confirmed the presence of components directly related to photosynthetic activity within the grape berry itself, in line with our results obtained by chlorophyll fluorescence analysis of the various berry tissues [18,23,28]. However, the precise role of this photosynthesis of berry tissues on the physiology and product quality of the grapes is still not understood.

In this work, we compared the metabolite profiles of the two photosynthetically active tissues (exocarp and seed) of berries from three developmental stages (green, *véraison* and mature) from the white grape variety cv. Alvarinho grown under contrasting microclimate conditions inside the grapevine canopy with respect to perceived light (HL or LL). One purpose of the present study was to link the observed differential photosynthesis of HL- and LL-grown berries [18], to possible differences in the tissue metabolite composition. In addition, we aimed to investigate the potential impact of two main short-term mitigation strategies applied in vineyards (i.e., foliar kaolin application and plant irrigation) on the metabolite profile of these grape berry tissues, in relation to their modulating effects on berry photosynthesis.

## 2. Results and Discussion

### 2.1. Global Effects on Grape Exocarp and Seed Metabolome

Untargeted metabolomics using liquid chromatography coupled with high resolution mass spectrometry (LCMS) was applied for comparing the composition of semi-polar compounds in the photosynthetically active berry tissues during their development, in both canopy light microclimates (LL and HL) and with both kaolin and irrigation as mitigation treatments. A large difference in the LCMS profiles was observed between exocarp and seed tissues (Figures S1 and S2 in Supplementary Materials). Considering this large difference, the mass peak alignment and subsequent data processing steps were separately performed for each tissue. We obtained the relative peak intensity data for a total of 395 and 398 putative metabolites detected in the exocarp (Supplemental File S1, Table S1) and seed (Supplemental File S2, Table S1), respectively, across all 128 samples analyzed.

**Figure 1:**
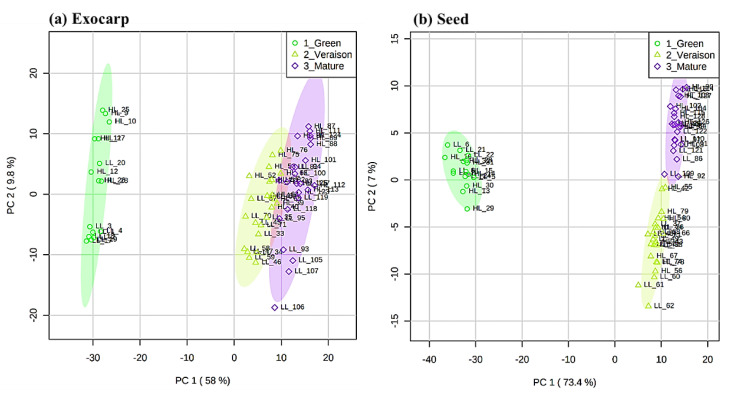
Principal component analysis (PCA) score plots of the liquid chromatography mass spectrometry (LCMS) metabolite data for the exocarp (**a**) and seed (**b**) in berries at three developmental stages (green, *véraison*, mature), including both microclimates (high light (HL) and low light (LL)) and both mitigation treatments. Colored ellipses represent 95% confidence interval (*n* = 4 for green stage and *n* = 3 for *véraison* and mature stages).

A principal component analysis (PCA), based on the relative intensities of the detected metabolites was performed for both tissues (Figure 1) in order to identify the main factors underlying the differentiation of the grape samples. Most of the total variability was explained by the first two PCs, i.e., 67.8% and 80.4% of the variation in the exocarp and in the seeds, respectively. For both tissues, PC1 was clearly related to the differences between the developmental stages, explaining 58.0% of the variance in the exocarp and 73.4% in the seed. In the exocarp (Figure 1a), the sample grouping was in line with their ripening order, i.e., green, *véraison* and mature stages, with the latter two stages being relatively similar compared to the green stage. In the case of the seed (Figure 1b), the *véraison* and mature stages were separated on PC2, explaining 7% of the variability, rather than on PC1. These results indicate that in both berry tissues, the metabolites detected by LCMS, i.e., mainly secondary metabolites, are mostly affected during the development from the green to *véraison* stage.

Analysis of Variance (ANOVA) in combination with false discovery rate (FDR) correction indicated that 362 metabolites in the exocarp (i.e., 91.6% of the metabolites detected by LCMS in this tissue) and 388 metabolites in seeds (i.e., 95.4% of the detected seed metabolites) differed significantly between the three developmental stages. A heatmap plot was constructed based on the 25 top-ranking metabolites according to the ANOVA test (Figure S3). For both tissues, the heatmap shows two main blocks of metabolites, i.e., a group of metabolites with higher abundance in the green stage and another group of metabolites higher in the later stages.

The FDR corrected *p*-values and the fold change (FC) values between the averages of the green and mature groups for both the exocarp (Supplemental File S1, Table S2) and seed (Supplemental File S2, Table S2) of untreated control samples grown in a HL microclimate (as an example) were calculated to select those compounds that were most influenced by ripening (PC1). In fact, 17.2% of the total of LCMS-compounds detected in the exocarp tissue appeared to be uniquely present (i.e., above detection threshold) in either green or mature grapes (Supplemental File S1, Table S2), while this was 19.8% in seeds (Supplemental File S2, Table S2). In addition, among the metabolites present at both developmental stages, 39.4% and 43.2% were significantly different (*p* < 0.05) between these developmental stages in the exocarp and seed, respectively. In the exocarp, a range of procyanidins (also called flavan-3-ols) were higher in green than in mature grapes (Supplemental File S1, Table S2), including procyanidin trimers (e.g., ID 308, 71-fold), dimers (e.g., ID 189, 4.5-fold and ID 140, epicatechin–gallocatechin, 17-fold) and the monomer catechin (ID 206, 4.2-fold), as well as stilbenes like resveratrol (ID 653, 58-fold) and piceid (ID 601, 2.8-fold). Their decrease upon ripening is in accordance with previous results with the exocarp of red grape berries [47,48]. Resveratrol in green berries has been suggested to play a role in preventing fungal infection and damage by UV irradiation [49]. On the other hand, a series of flavonol glycosides significantly increased upon ripening, such as quercetin 3-*O*-glucoside (ID 492, 5.4-fold), kaempferol-3-glucoside (ID 545, 46-fold) and an isorhamnetin-hexoside (ID 583, 49-fold), in accordance with previous results obtained with the skin of both white and red grapes [50]. In the seed (Supplemental File S2, Table S2), there was a similar decrease in various procyanidins, such as gallocatechin (ID 229, 180fold) and other polymeric compounds from the same class (e.g., ID 150, 28-fold; ID 377, 19-fold; ID 579, 12-fold), which is in accordance with previous results with seeds of red grape berries [47]. On the other hand, seed ripening coincided with an increase in resveratrol (ID 979, 7.2-fold) and several of its putatively-identified oligomers including a dimer (ID 926, 36-fold), a trimer-hexoside (ID 990, 33-fold) and a tetramer (ID 1074, 169-fold).

**Figure 2:**
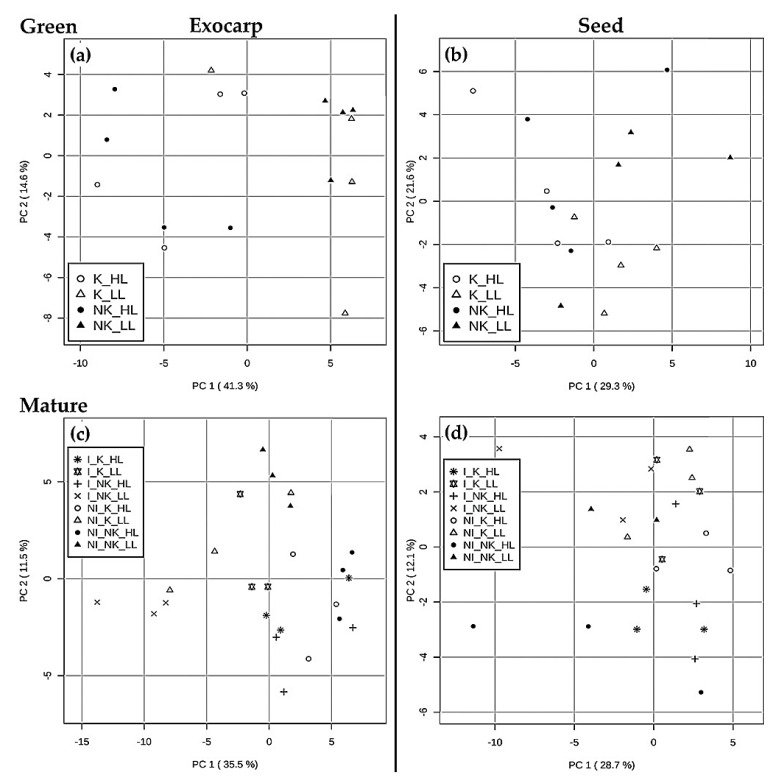
Principal component analysis (PCA) score plots based on the LCMS metabolite data for both exocarp (**a**,**c**) and seed (**b**,**d**) at the two most contrasting ripening stages (**a**,**b**—green and **c**,**d**—mature), including all microclimates and treatments (*n* = 4 for green stage and *n* = 3 for mature stage). The abbreviations in the legend represent: NI—Non-Irrigation; I—Irrigation; NK—Non-kaolin; K—Kaolin; LL—Low Light microclimate; HL—High Light microclimate.

Subsequently, PCA was performed for each developmental stage and for each grape berry tissue separately (Figure 2 and Figure S4). For the PCA of the green stage, only the effects of microclimates and foliar kaolin application could be assessed, since at this developmental stage, no irrigation was yet applied to the vineyards. At the green stage, the PCA result plots indicated a separation of samples mainly according to the canopy microclimate (LL vs. HL), most specifically in exocarp samples (PC1 explaining 41.3% and 29.3% of total variance in exocarp and seed, respectively), while no clear sample grouping was observed for kaolin-treated versus untreated plants in either tissue, neither based on the first two PCs (Figure 2a,b) nor upon considering subsequent PCs (PC3 and PC4; data not shown). At the *véraison* stage (Figure S4), the berry exocarp metabolome (Figure S4a) was mainly influenced by microclimate as well (PC1, 24.9%) and secondly also by irrigation (PC2, 18.9%). In contrast, at this developmental stage, the seed metabolome (Figure S4b) was mainly affected by the irrigation treatment (PC1, 38.0%). At the mature stage, the exocarp metabolome (Figure 2c) was primarily influenced by the canopy microclimate (PC1, 35.5%) and secondly by the irrigation treatment (PC2, 11.5%), while for mature seeds (Figure 2d) no clear grouping of the differently treated berries was detected.

To assess the composition of primary metabolites in exocarps, gas chromatography mass spectrometry (GCMS) analysis was performed. This was done for mature berries only, since this stage is most directly related to the quality of grapes and wine. The unbiased processing of the 24 exocarp samples resulted in the relative abundances of 99 metabolites, mainly sugars, amino acids and organic acids (Supplemental File S1, Table S3, Figure S5). In contrast to the PCA based on LCMS metabolites (Figure 2c), the PCA based on these GCMS compounds did not reveal clear effects of either microclimate, kaolin or irrigation on the metabolic composition of these mature exocarp samples (Figure S6). The lack of irrigation effects suggests that the accumulation of primary compounds is unrelated to the effect of irrigation on the photosynthetic activity in these mature exocarps [18]. 

ANOVA Simultaneous Component Analysis (ASCA) was subsequently used to determine which of the growth conditions, as well as their possible interactions, exerted a significant effect on the metabolome of the exocarp and seeds at each of the three berry developmental stages, based on either the LCMS and GCMS analysis (Table 1). In addition, we applied a N-way ANOVA to study the effect of the growth conditions on each metabolite in more detail (Table 1: numbers between brackets indicate numbers of significant metabolites). The significant compounds (all, or top 20) following from the ANOVA analysis were subsequently manually annotated (for exocarp—Supplemental File S1, Tables S4–S9; for seed—Supplemental File S2, Tables S3–S5).

It is worth noting that the *p*-values of the univariate tests were adjusted for multiplicity by the Benjamini–Hochberg false discovery rate (FDR) procedure to control (in expectation) the proportion of false positive differential metabolites. Nevertheless, there is still a chance for false positive results, due to a relatively large number of variables of both the treatments tested and metabolites detected compared to the low number of biological replicates per group sample. Therefore, in the subsequent part we only focus on those significantly differing metabolites with the lowest *p*-values and for which the size of the effect (i.e., the fold change) was much larger than the overall technical variation for that specific compound (as determined from the quality control samples) (Supplemental Files S1 and S2, Table S1).

This ASCA approach identified the berry microclimate as the main growth condition influencing the LCMS-metabolites in both berry tissues at all three developmental stages, except for seeds at the *véraison* stage (Table 1), which is in accordance with the PCA results based on these LCMS-metabolites (Figure 2). In addition, the ASCA results for the GCMS-metabolites in the mature exocarp indicated that the irrigation treatment itself has no significant impact (cf. Figure S6); in contrast, here the berry microclimate has a significant impact, which was undetectable by the PCA model (Figure S6). Soil irrigation had a significant impact on the LCMS profiles: for exocarp at both *véraison* and mature stages and for seeds at the *véraison* stage only. In contrast, kaolin did not significantly influence the LCMS-profiles at either developmental stage or tissue, while it did significantly impact the GCMS profiles in the mature exocarp, but no significant differences could be shown for the individual metabolites. Previous studies with red grape varieties showed that kaolin application had a positive influence on both phenylpropanoids and flavonoids [21], while it only had a minor effect on both free and bound volatile organic compounds in the berries [51]. 

Our ASCA models also showed a significant interaction effect between irrigation and kaolin on both LCMS and GCMS compounds in the berry exocarp, with the ANOVA model indicating a few significantly differing compounds in the GCMS-profiles only. The size of this interactive effect on individual GCMS compounds was rather small, i.e., less than 40% change in abundance (Supplemental File S1, Table S4), while the direction of this effect differed between compounds: kaolin application reduced the irrigation-induced increase and decrease in l-alanine and quininic acid, respectively, while the (small but significant) increases in the abundance of several sugars induced by either kaolin or irrigation alone were counteracted when both treatments were applied together (Figure S7). Previous studies using whole red grape berries did not observe any significant interactive effect of kaolin and irrigation on free and bound volatile compounds [51], or total soluble solids, total organic acids, anthocyanins and phenolics [52]. Altogether it seems likely that the interaction of these two mitigation treatments does not exert a large, if any, effect on the global metabolome of mature grapes in practice.

#### 2.1.1. Specific Effects of Microclimate

**Figure 3:**
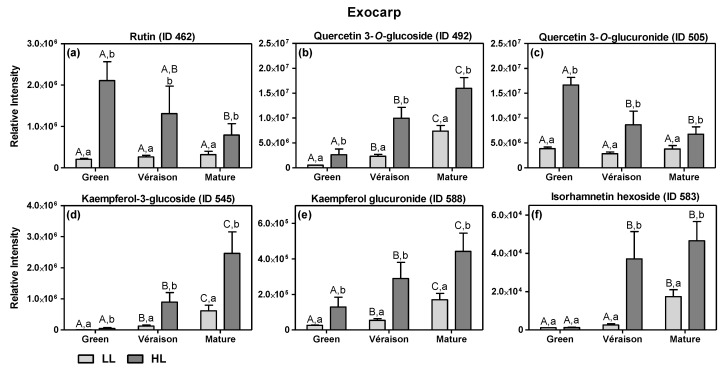
Relative intensities of the six main flavonol compounds as detected by LCMS (means and SD of *n* = 8–12) for exocarp tissue at two canopy microclimates (LL and HL; independent of mitigation treatment) and at three developmental stages (green, *véraison* and mature). The flavonols considered are: (**a**) rutin (quercetin-3-*O*-rutinoside) (ID 462), (**b**) quercetin 3-*O*-glucoside (ID 492), (**c**) quercetin 3-*O*-glucuronide (ID 505), (**d**) kaempferol-3-glucoside (ID 545), (**e**) kaempferol glucuronide (ID 588) and (**f**) isorhamnetin hexoside (ID 583) (Supplemental File S1, Tables S5–S7). Statistical analysis (two-way ANOVA, *p* ≤ 0.05) was applied after data Log_2_ transformation. Statistical notation above the bars: the capital letters refer to differences between developmental stages for the same microclimate, while the lowercase letters refer to differences between the two light microclimates for each stage.

By comparing the fold change (FC) values of the average metabolite abundances in the HL and LL groups, i.e., the intensity ratio between the average of the HL and LL samples irrespective of mitigation treatment, we identified those metabolites that were most strongly affected by microclimate (for exocarps—Supplemental File S1, Tables S5–S7; for seeds—Supplemental File S2, Tables S3 and S4). Overall, the HL exocarps were characterized by a consistently higher level of several flavonol conjugates (Figure 3) (Supplemental File S1, Tables S5–S7), except for isorhamnetin hexoside at the green stage (Figure 3f). During berry development, the flavonol conjugates showed differential accumulation patterns (Figure 3). On the one hand, the relative intensities of quercetin-3-*O*-rutinoside (rutin) and quercetin 3-*O*-glucuronide decreased in HL exocarps during development, while in LL, exocarps kept their values constant (Figure 3a,c). The remaining flavonols showed an increase in intensity during development for both microclimates (Figure 3b,d–f). These results suggest that there was a development-specific flavonol composition and that this was significantly influenced by the light microclimate. If the relative abundance values of these six compounds are added up, notwithstanding their potentially differential ionization efficiencies in the MS source, the mean value of the total of these flavonols are significantly higher in HL-exocarps than in LL ones at all developmental stages (Figure S8a). These results suggest that HL berries had their maximum level of total flavonols already at the green stage and this high level was maintained upon subsequent ripening; in contrast, during ripening of LL berries, their flavonol content was continuously increasing to a level that at the mature stage was still lower than that of HL berries. By using a calibration curve of authentic standard, absolute quantities of the main flavonol quercetin 3-*O*-glucoside were obtained (Figure S8b), and these absolute values showed the same pattern as the relative peak values (Figure 3b).

The increase in flavonols by HL compared to LL is in accordance with previous reports on the microclimate effects on both white grape berries [42,53,54] and red varieties [25,55]. This increase, especially relevant in the green stage when the total amount of flavonols peaks in exposed clusters, had a parallel with the increased photosynthetic activity of exocarps under HL conditions in the green stage [18]. Flavonols are generally considered to have antioxidant and/or “sunscreen” abilities, thereby protecting the photosynthetic apparatus as well as other macromolecules from excess solar radiation in situ [56]. Thus, the higher levels of flavonols in HL exocarps may represent an acclimation response to the higher intensity light microclimate, possibly to protect their photosynthetic system from radiation-mediated oxidative damage [54], and therefore keeping its photosynthetic activity until the later stages of development [18].

In HL exocarps, we also observed higher levels of some putatively annotated glycosylated aroma compounds, such as a vanillyl alcohol hexoside (ID 136) and geraniol-hexose-pentose (ID 685), as compared to LL berries (Supplemental File S1, Tables S6 and S7).

**Figure 4:**
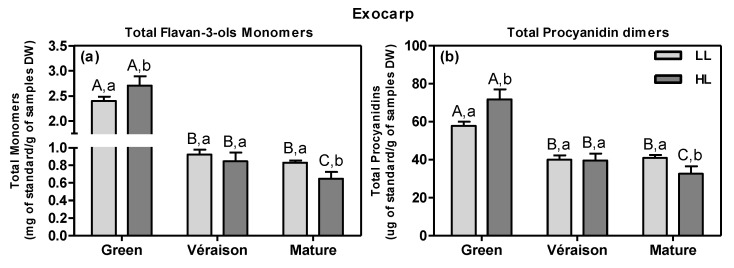
Total flavanols in exocarp tissue at two canopy microclimates (LL and HL; independent of mitigation treatment) and at three developmental stages (green, *véraison* and mature). (**a**) Total flavan-3-ols monomers levels (mg/g of dry weight (DW)): catechin, epicatechin, epicatechin-3-*O*-gallate. (**b**) Total procyanidin dimer levels (µg/g DW): procyanidin B1, B2 and B3. Statistical analysis with two-way ANOVA (*n* = 8–12, +SD, *p* ≤ 0.05). Statistical notation is the same as in Figure 3.

Another class of grape flavonoids, the flavan-3-ols or procyanidins, comprising both monomers and a range of oligomers/polymers of (epi)catechin and (epi)gallocatechins, are key to wine quality as they confer astringency and bitterness [57]. In addition, they protect the plant and its fruits against pathogens, pest insects and herbivores [58]. Absolute quantities of six selected flavan-3-ols were obtained by using calibration curves of authentic standards; since their abundance patterns were more or less similar across samples (data not shown), their levels were added up to calculate total monomers and total procyanidins (Figure 4). At the green stage, HL berry exocarps had significantly higher contents of total flavan-3-ols, i.e., both (epi)catechin monomers (Figure 4a) and procyanidin dimers (Figure 4b), compared with LL ones (Figure 4a). During ripening, these contents decreased in both microclimates, but more so in HL than in LL. In addition, the HL microclimate led to an up-regulation of several other compounds including flavan-3-ols in the berry exocarp, when compared to LL; at the green stage, the HL exocarps contained more hydroxy-procyanidin trimers (e.g., ID 147 and ID 151) and a procyanidin conjugate (e.g., ID 310) (Supplemental File S1, Table S5). However, at the mature stage, the flavan-3-ols monomers, e.g., (+)-catechin (ID 206), dimers e.g., procyanidin B1 (ID 174) and trimers, (e.g., ID 215) were lower in HL exocarps (Supplemental File S1, Table S7). The analysis by GCMS confirmed that mature exocarps accumulated less catechin monomers in HL than in LL conditions (ID 13276, FC HL/LL = 0.8). 

There are yet unexplained and conflicting results reported on the influence of light on the accumulation of flavan-3-ols in white grape berries. On the one hand, one study showed that the total flavan-3-ol content was affected neither by shading treatments nor by more incoming light due to leaf removal [53]. On the other hand, at the green stage, shaded white grapes contained more total flavan-3-ols monomers than exposed ones [42], while in another study with two different white varieties, the amount of various flavan-3-ols was greater in sun-exposed berries than in shaded ones [59], in line with our results at the green stage (Figure 4). However, in the skin of red mature grapes, shading resulted in a decrease in flavan-3-ols monomers and, subsequently, in a decreased level of condensed tannins (procyanidins) [60]; these results are in contrast to those previously obtained with white grapes and presented here for LL mature berry exocarps [42] (Figure 4). 

**Figure 5:**
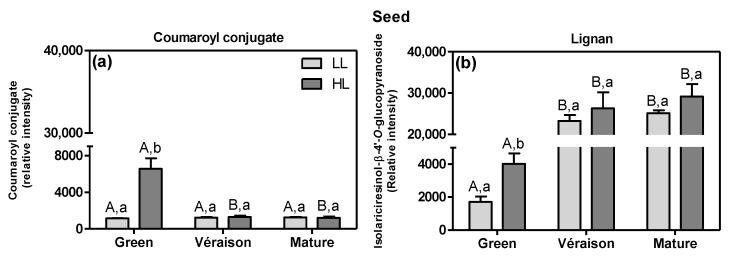
Examples of seed hydroxycinnamic acids, (**a**) a coumaroyl conjugate (ID 285) and (**b**) a lignan type—isolariciresinol-β-4′-*O*-glucopyranoside (ID 613) detected by LCMS (mean values + SD, *n* = 8–12) significantly differing between canopy microclimates (LL and HL; independent of mitigation treatment) and at three developmental stages (green, *véraison* and mature). Statistical analysis (two-way ANOVA, *p* ≤ 0.05) was applied after data Log_2_ transformation. Statistical notation is the same as in Figure 3.

The microclimate also affected the metabolic profile of seeds, at both green and mature stages (Supplemental File S2, Tables S3 and S4). At the green stage, seeds from HL showed an up-regulation of hydroxycinnamic acid compounds, i.e., upstream from the flavonoid pathway (Supplemental File S2, Table S3), including a coumaroyl conjugate (ID 285, FC HL/LL = 4.5) and the lignan-type hydroxycinnamic acid dimer isolariciresinol-β-4′-*O*-glucopyranoside (ID 613, FC = 2.2) (Figure 5). Hydroxycinnamic acids including lignans have been shown to possess antioxidant activity and are associated with the biosynthesis of lignins [61], which are key in seed lignification [62]. In our experiments, the level of the lignan isolariciresinol increased from the green to *véraison* stage and then its abundance was maintained up to the mature stage (Figure 5b); this pattern is in agreement with the degree of lignification of grape seeds [63].

The HL microclimate, as compared to LL, also led to a higher relative abundance of compounds from the flavonoid pathway itself, specifically flavan-3-ols in seeds at the green stage, including a procyanidin pentamer (ID 317, FC = 2.2), a (epi)gallocatechin-conjugate (ID 162, FC = 1.7), a pentahydroxyflavan dimer (ID 170, FC = 1.6) and the gallocatechin (ID 229, FC = 1.5) (Supplemental File S2, Table S3). In contrast, mature HL seeds accumulated less stilbene derivatives, such as (+)-alpha-viniferin-hexoside (ID 990, FC = 0.8) and viniferin 3″-glucoside (ID 1027, FC = 0.7) (Figure S9). Stilbenes are an effective response against pathogen infection and abiotic stress and contribute to the final nutraceutical quality of both seeds and wine [49,64,65]. To our knowledge, the effect of light conditions on stilbenes in grape seeds has not been reported before.

#### 2.1.2. Specific Effects of Irrigation

**Figure 6:**
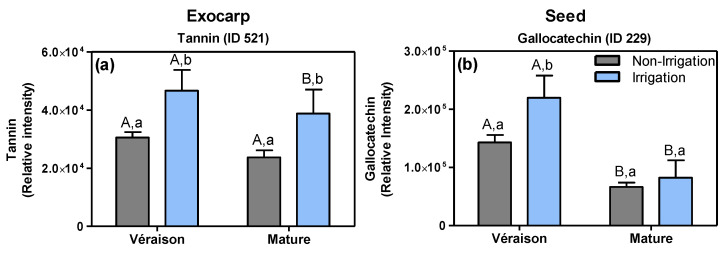
Relative abundance of (**a**) a tannin (ID 521) in the exocarp and (**b**) for gallocatechin (ID 229) in the seed, as obtained by LCMS analysis (means + SD, *n* = 8–12). Grape berries were grown under non-irrigation (grey bars) and irrigation conditions (blue bars) and collected at two developmental stages (*véraison* and mature). Data of HL and LL berries, combined. Statistical test: two-way ANOVA, *p* ≤ 0.05. Statistical notation above the bars: the capital letters refer to differences between developmental stages for the same treatment condition, while the lowercase letters refer to differences between the control and irrigation treatment, within each developmental stage.

The irrigation of the soil resulted in an up-regulation of thonningianin B (ID 521, putatively identified), a tannin-type of compound, in the exocarp at both the *véraison* and mature stage (Figure 6a) (Supplemental File S1, Tables S8 and S9). In seeds, at the *véraison* stage, the irrigation led to an accumulation of several flavan-3-ols such as gallocatechin (ID 229, Figure 6b) and an (epi)gallocatechin-conjugate (ID 162) (Supplemental File S2, Table S5). Additionally, at the *véraison* stage, the irrigation resulted in a down-regulation of primary metabolites, including: d-fructose 1,6-bisphosphate (ID 79) and arginine (ID 77) in exocarps (Figure S10a,b, Supplemental File S1, Table S8) and both a hexose sugar (ID 21) and the phenylpropanoid coutaric acid (ID 315) in seeds (Figure S10c,d, Supplemental File S2, Table S5). On the other hand, in exocarps at the mature stage, the irrigation treatment resulted in a lower accumulation of a hydroxy-procyanidin trimer (ID 151), a (epi)catechin-gallocatechin dimer (ID 122) and (+)-gallocatechin (ID 131) (Supplemental File S1, Table S9).

Genebra et al. [66], likewise, showed that seeds from irrigated grapevines at full maturation had higher flavan-3-ols and tannins contents compared to non-irrigated ones, which was explained by a slower berry ripening upon irrigation. In fact, Castellarin et al. [67] argued that water deficiency accelerates the ripening of grape berries. In another study, Koundouras and collaborators [68] showed that the total amount of flavan-3-ols in the seed (per fresh weight) was higher in fully irrigated vines compared to non-irrigated ones, likely due to the effect of the more vigorous canopy growth on the berry microclimate. In addition, the skins and seeds of berries from fully irrigated plants of the red Syrah variety tasted more astringent than those from non-irrigated ones, which was attributed to the higher levels of various flavan-3-ol-type of polyphenols [69]. The present and previous studies thus indicate that irrigation can delay berry ripening and thereby result in the ripening-dependent decrease in flavan-3-ols (e.g., Figure 4 and Figure 6), which may have an effect on quality traits of both grape seed and wine.

### 2.2. Changes in Carbon Skeletons

**Figure 7:**
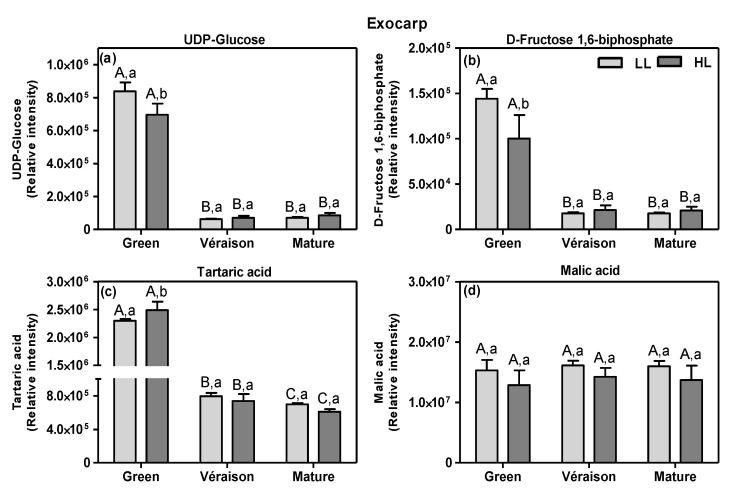
Relative intensity of some sugars (**a**,**b**) and organic acids (**c**,**d**) detected by LCMS (means + SD, *n* = 8–12) in exocarps of grapes grown in two light microclimates (LL and HL; independent of mitigation treatment) and harvested at three developmental stages (green, *véraison* and mature). (**a**) UDP-glucose (ID 97), (**b**) d-fructose 1,6-biphosphate (ID 79), (**c**) tartaric acid (ID 168) and (**d**) malic (ID 51) acid. See Figure 3 for statistical information.

Berries are strong sinks importing massive amounts of photoassimilates/carbon structures, mainly sucrose, from the leaves [70]. Since exocarps and seeds are both photosynthetically active, especially in the green stage of berry development [18,23,28], they may locally contribute by supplying energy and carbon-skeletons needed for the biosynthesis of compounds accumulating in these berry tissues. The heatmap plot based on LCMS metabolites (Figure S3) showed that specific sugars and organic acids were relatively high at the green stage and decreased with ripening in either or both exocarps and seeds, including UDP-glucose (ID 97) and tartaric acid (ID 168) in the exocarp (Figure 7) and malic acid (ID 55) in the seed (not shown).

UDP-glucose and d-fructose 1,6-biphosphate accumulate mainly at the green stage, especially in LL grapes, decreasing upon subsequent further grape berry development (Figure 7a,b). The result for UDP-glucose during development is in accordance with a previous study with whole red grape berries [44]. Moreover, it was also shown that d-fructose 1,6-biphosphate was low at the green stage, peaked at the *véraison* stage, and afterwards it sharply decreased until the end of grape berry development [44].

The ripening-dependent decline in tartaric acid (Figure 7c) is consistent with previous reports [40,43]. Moreover, its level was significantly higher in green HL exocarps than in LL ones, which may be due to the higher photosynthetic activity of HL berries [18,23]. The high amounts of organic acids endow green fruits with a sour taste for defense against herbivores [71], while in mature berries, they are essential for both wine production, as they protect the fermentation process from bacterial contamination [72], and wine taste. In contrast to tartaric acid, malic acid was more or less constant in exocarps throughout ripening (Figure 7d). The fact that malic acid did not decrease, as previously demonstrated for whole berries [43,73], suggests the tissue-specific effects of ripening on this organic acid. Here, we also show that malic acid did not significantly differ between the two light microclimates at any stage (Figure 7d), irrespective of their differential berry photosynthetic activity [18], suggesting that malic acid accumulation in the exocarp occurs mainly through the metabolism of sugars translocated from leaves to the berries, rather than from fruit photosynthesis itself. Clearly, further studies are needed to determine the exact role of berry in situ photosynthesis in the biosynthesis of organic acids, since a previous study indicated a positive relation [73], but together, these results seem to point toward a role in the biosynthesis of specific organic acids. While the biosynthesis and catabolism of malic acid in grape berry is well established, being mainly produced from unloaded photoassimilates and accumulated in the vacuoles of mesocarp cells at the green phase [73], fewer studies are available for tartaric acid. However, it is known that the main pathway for the synthesis of its precursor, ascorbate (vitamin C), is fueled by carbon derived from photosynthesis [74]. 

### 2.3. Lipid-Soluble Antioxidants and Lipid Oxidation in Photosynthetically-Active Grape Tissues

**Figure 8:**
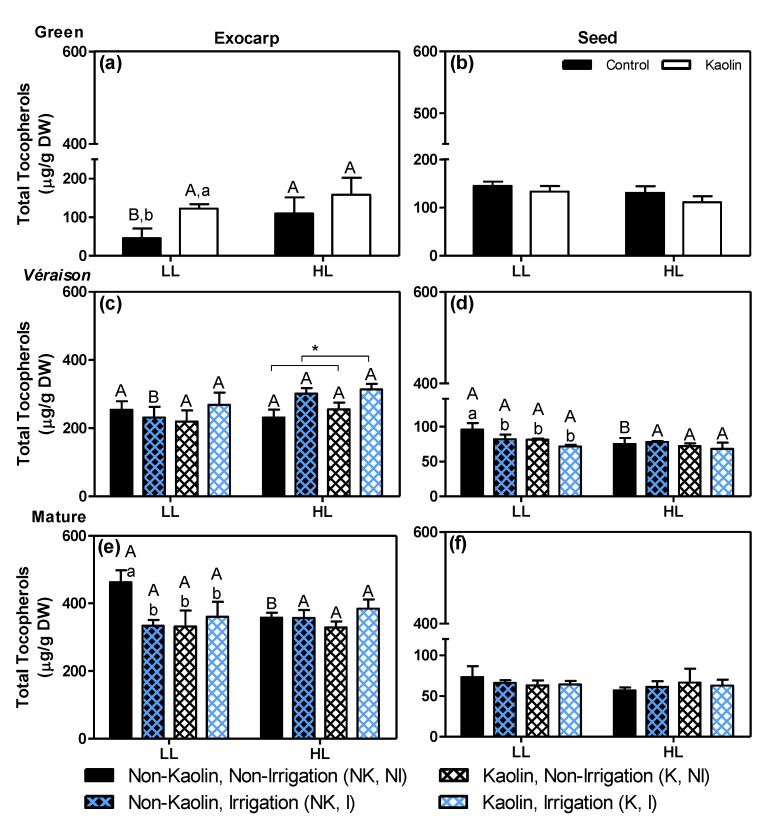
Total tocopherol contents (sum of the three main tocopherols detected, mean values + SD of *n* = 3 or 4) in exocarps (**a**,**c**,**e**) and seeds (**b**,**d**,**f**) of berries grown in either low light (LL) or high light (HL) microclimate conditions in the canopy. Kaolin (K) or no kaolin (NK) was applied to the plant leaves before fruit set; after green berries were developed, plants were either irrigated (I) or non-irrigated (NI). Samples were collected at three development stages: green (**a**,**b**), *véraison* (**c**,**d**) and mature (**e**,**f**). Statistical notations above the bars: at each developmental stage, the capital letters refer to differences between the two light microclimates within the same treatment, while the lowercase letters refer to differences between treatment combinations within the same light microclimate (bars with no or a common letter indicate no significant differences; two-way ANOVA, *p* ≤ 0.05). Notation with an asterisk means that only one factor (in this case the irrigation) was significant.

Tocopherols (vitamin E) are effective lipid-soluble antioxidants that can neutralize various reactive oxygen species, thereby protecting vulnerable membranes, such as chloroplast thylakoids and the photosynthetic apparatus, from photooxidative damage [75]. The main tocopherol species detected in both exocarps and seeds was α-tocopherol (Figure S11) followed by its γ-form (Figure S12), while δ-tocopherol was a minor compound (Figure S13) and β-tocopherol (not shown) was not detectable at all. As the effects of both microclimate and mitigation strategies were more or less similar on these three detected tocopherol species (i.e., α-, γ- and δ-), in each tissue, we added up their levels (Figure 8).

In green berries (Figure 8a,b) the exocarp and seed contain about similar total tocopherol levels, with a significant effect of the microclimate on the exocarp of untreated plants (Figure 8a; HL/LL = 2.42), as well as a significant effect of kaolin on the LL-exocarps (K/NK = 2.71). During ripening, the tocopherol content of the LL-exocarp controls increased about 10-fold as compared to their green stage, while that of the HL-exocarp controls increased less, i.e., about 3.27-fold, and at the mature stage reached lower levels than LL ones (Figure 8a,c,e; black bars). In line with this elevated tocopherol level in green HL-exocarps, a previous study with Sauvignon Blanc grapes at the green stage indicated that elevated light exposure induced the expression of tocopherol biosynthetic genes, which subsequently leads to the accumulation of lipophilic antioxidants, presumably to maintain the cellular redox balance and to protect the photosynthetic machinery from light stress [42,76]. In addition, the stimulating effect of kaolin on tocopherol content in green LL-exocarps is in line with previously reported elevated levels of both chlorophylls and carotenoids [18], and is likely due to the fact that more light can reach the inner parts of the canopy by the increased reflection [18].

In seeds, there were no relevant effects by either the light-microclimate or mitigation treatments, and in contrast to the marked increase in tocopherols in exocarps, a decrease of about 50% was observed from the green to mature stage (Figure 8b,d,e). In green seeds, tocopherols may play an antioxidant role, preventing lipid peroxidation in both the thylakoid membranes, which are photosynthetically active [18], and in the developing embryo and endosperm; in mature seeds, it is likely mainly present in the endosperm where it may play an important antioxidant role during seed storage and germination [77]. 

**Figure 9:**
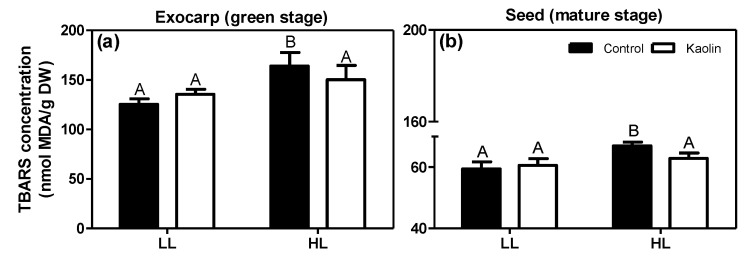
Levels of thiobarbituric acid-reactive-substances (TBARS; in nmol MDA/g DW) in green exocarps (**a**) and mature seeds (**b**). Grape berries were from LL and HL microclimates and from plants with or without (control) foliar kaolin application. Different capital letters above the bars refer to significant differences between microclimates within the same treatment (*p* ≤ 0.05; *n* = 3 or 4).

The effects of the microclimate and kaolin on lipid peroxidation and lipid-breakdown products in the berry tissues were assessed using the thiobarbituric acid-reactive-substances (TBARS) assay. Due to the limited amount of available material, only exocarps from green berries and seeds from mature berries, both from non-irrigated plants, could be analyzed (Figure 9). In both tissues from non-kaolin treated control plants, there was a significant increase in TBARS in the HL berries as compared to the LL ones, suggesting enhanced lipid peroxidation due to photo-oxidative stress in the HL berries. This photo-oxidative stress effect may lead to photosynthetic impairment [78], while in the mature seeds it may affect the lipid composition and thus the quality of the seed and its derived products [79]. The kaolin treatment prevented the increase in the amount of TBARS in both tissues of HL-grown berries, suggesting a reduction in photo-oxidative damage, specifically in the direct-light exposed (HL) grapes. Indeed, previous studies likewise showed that kaolin-sprayed grapevines had lower TBARS levels compared to control ones, in both berries and leaves [20,80].

Specific antioxidant mechanisms present in the exocarp may prevent or decrease this photo-oxidative stress in HL berries. Indeed, during berry development, the exocarp exhibited an increase in tocopherols (Figure 8a,c,e) and total flavonols (Figure S8a). In contrast, the total carotenoids content decreased during exocarp ripening [18]. Thus, carotenoids may be involved in the photoprotection of exocarp cells in the early (green) stages of berry development only, while both tocopherols and flavonols can act together upon subsequent ripening. Moreover, some of these ROS scavengers can support the non-photochemical quenching (NPQ) and may also compensate for the previously observed decrease in NPQ efficiency during berry ripening [18,76].

### 2.4. Effects on Grape Quality-Related Compounds: Total Phenolics and Sugars in Mature Fruit 

**Figure 10:**
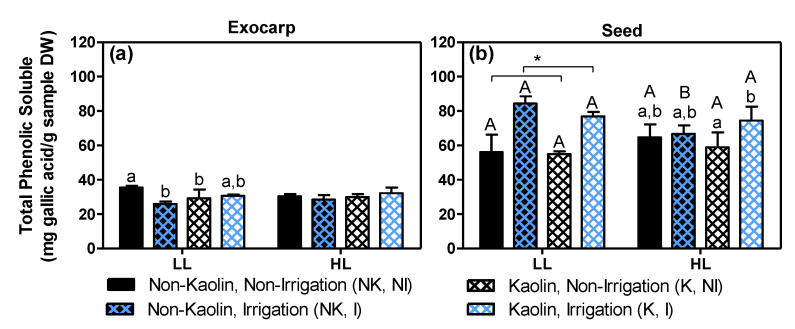
Total phenolic contents (mg gallic acid/g sample DW) in exocarps (**a**) and seeds (**b**) from mature stage berries. Growth conditions and treatments are the same as in Figure 8. Statistical notations above the bars: the capital letters refer to differences between the two light microclimates within the same treatment, while the lowercase letters refer to differences between treatment combinations within the same light microclimate (bars with no or a common letter indicate no significant differences; two-way ANOVA, *p* ≤ 0.05). Notation with an asterisk means that only one factor was significant (here: irrigation in the case of LL seeds).

In grape berries, the majority of phenolic compounds, a key parameter for wine quality, are located in the exocarp and seeds [33,34]. As the untargeted LCMS analysis indicated significant effects on various phenolic compounds including flavonoids in both exocarps and seeds (e.g., Figure 3, Figure 4 and Figure 6; see also Supplemental Files S1 and S2), we subsequently applied the Folin–Ciocalteu colorimetric method to determine the total amount of soluble phenolic compounds (Figure 10).

Overall, the total phenolic content was about two times higher in seeds than in exocarps. In exocarps (Figure 10a), neither a significant effect by microclimate nor a significant effect by either mitigation treatments in HL berries was observed, while in LL berries, both mitigation treatments and especially irrigation resulted in a decrease in total phenolics. This effect of irrigation is in accordance with previous studies showing an up-regulation of the grape flavonoid pathway by water deficiency [67,81]. In seeds (Figure 10b), this irrigation treatment induced a significant increase in total phenolics in LL-grapes, irrespective of kaolin treatment, while in HL grapes this increase by irrigation was only observed for kaolin-treated grapevines. It has been reported that water deficiency causes an up-regulation of the flavonoid pathway in both skins (=exocarp) and whole berries [67,81]. Our results (Figure 10) and previous ones [69], showed that in seeds, the opposite occurs, i.e., the content of phenolic compounds increases by irrigation. Apparently, water deficiency exerts opposite effects on the (poly)phenolic pathways in seeds versus other berry tissues.

**Figure 11:**
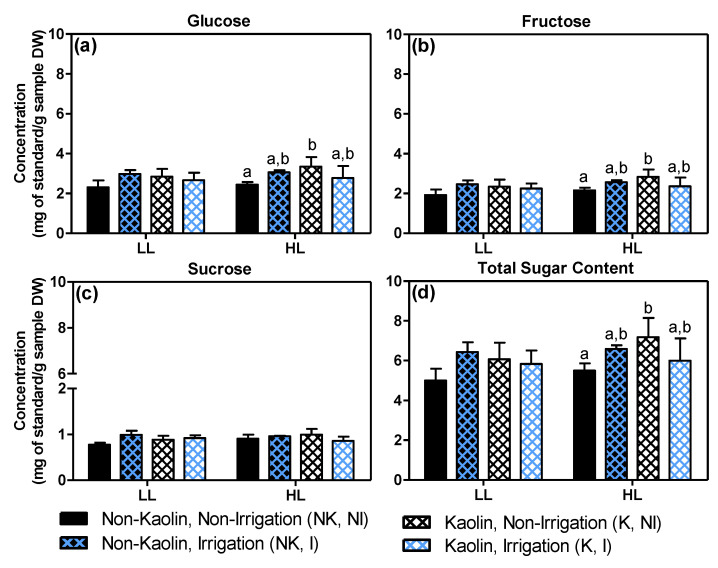
Levels of main sugars (in mg/g sample DW, mean values *n* = 3–4, +SD) in the exocarp of mature berries grown in either low light (LL) or high light (HL) microclimate conditions in the canopy: (**a**) glucose, (**b**) fructose, (**c**) sucrose and (**d**) their summed values, in this paper referred to as total sugar content. Kaolin (K) or no kaolin (NK) was applied to the plant leaves before fruit set; plants were irrigated (I) or non-irrigated (NI) after the green stage. Statistical notations above the bars: the lowercase letters refer to differences between treatment combinations within the same light microclimate (values with a common letter or no letter at all, indicate no significant differences; two-way ANOVA, *p* ≤ 0.05).

The major sugars present in ripe grapes are glucose and fructose, which both result from sucrose translocated from the leaves [82], while others are produced from the accumulated malate [73]. Previous studies dealing with the influence of foliar kaolin on sugar biosynthesis in leaves showed that this application led to an increase in a vast array of leaf primary metabolites, including sucrose, glucose and fructose, as well as several organic acids [17]. However, there is yet no information regarding sugar contents in relation to kaolin or other mitigation effects in the grape exocarp, which is photosynthetically active, specifically. Figure 11 shows the levels of the three main individual sugars, as well as their summed values (i.e., here referred to as total sugar content) in the exocarp of the mature grapes.

Under untreated control conditions (NK, NI), HL exocarps had sugar levels, similar to LL exocarps, while with kaolin only (K, NI), they contained significantly more glucose (Figure 11a; +37%) and fructose (Figure 11b; +32%), and consequently 30% more total sugars (Figure 11d). Since the mature exocarps of HL and LL berries showed similar photosynthetic capacities [18], we assume that this positive effect of kaolin on exocarp sugar content is related to the higher sugar biosynthesis in the grape leaves, leading to a higher import into the berries, including the exocarp, and/or to the higher biosynthesis from the accumulated malate that is used to produce sugars from the *véraison* stage, onwards.

## 3. Materials and Methods

### 3.1. Grapevine Field Conditions and Sampling

Grape berry samples were collected from Alvarinho cultivar grapevines (*Vitis vinifera* L.) grown in a field trial conducted in 2018 in the organic vineyard Quinta Cova da Raposa in the Demarcated Region of Vinho Verde, Braga, Portugal (41°34′16.4″ N 8°23′42.0″ W). Details concerning the vineyard and treatments are described in Garrido et al. [18]. Briefly, all treatments—kaolin (K) and non-kaolin (NK) application on leaves, and irrigation (I) and non-irrigation (NI) of grapevines—were applied in a complete factorial design in two blocks, each with three to four vines per treatment. The kaolin suspension (5% *w*/*v* in water) was applied on both 6 July and 27 July 2018, corresponding to four and seven weeks after anthesis (WAA), respectively. Irrigation of half of the plants started on 26 July (seven WAA) by means of drip irrigation (one dripper per vine with an average capacity of 5.5 ± 1.6 L h^−1^) for 2 h every three days. Grape clusters with contrasting light exposures in the canopy, called low light (LL) and high light (HL) microclimates, were collected randomly at each experimental condition and at three distinct developmental stages; green (16 July, 6 WAA), *véraison* (29 August, 12 WAA), and mature (17 September) berries were immediately frozen in liquid nitrogen and stored at −80 °C. The exocarp (=skin) and seeds were isolated from the whole frozen grape berries. Firstly, the berry was broken with a slight impact of a pestle in a mortar (both pre-cooled with liquid nitrogen), which allowed us to isolate the seeds. Secondly, the exocarps were dissected from the remaining frozen berry fragments in a petri dish placed in a box with ice, and then quickly transferred to a liquid nitrogen cooled falcon tube. Finally, both seeds and exocarp pieces were ground to a fine powder, using a mortar, a pestle and liquid nitrogen, and freeze-dried for 48 h before metabolomic analyses. At each ripening stage, we sampled berries from 3 (both *véraison* and mature) or 4 (green) biological replicates for each condition, in which 1 replicate resembled a mix of 5 to 10 berries from 3 to 5 clusters from 6 to 8 plants, resulting in a total of 128 samples. All dried samples, conditioned in boxes with silica to maintain the dehydration, were transported to Wageningen, the Netherlands, in order to analyze them by complementary targeted and untargeted metabolomics platforms.

### 3.2. Untargeted Metabolomics by Liquid Chromatography Mass Spectrometry (LCMS) and Gas Chromatography Mass Spectrometry (GCMS)

#### 3.2.1. LCMS Analysis

All 128 samples were used and extracted according to De Vos et al. [83]. Quality control (QC) samples (*n* = 5, per each batch analysis) were also prepared with a mix of the grape berry tissues in order to estimate the overall technical variation per compound. In short, 20 mg dry weight (DW) grape berry tissue was transferred to 2 mL plastic safe-lock Eppendorf tubes and extracted with 600 μL of 75% (*v*/*v*) methanol/water + 0.1% formic acid (FA). After vortexing (10 s) and sonication (15 min) (these steps were performed twice), samples were centrifuged (16,100× *g*) for 15 min and the supernatant was collected. Chromatographic separation (5 µL of in injection) was performed on an HPLC system (Waters Acquity, Milford, MA, USA) with a C18 column (Phenomenex Luna 150 × 2 mm i.d., 3 µm—Torrance, CA, USA) using ultra-pure water (eluent A) and acetonitrile (eluent B) both acidified with 0.1% FA at a flow rate of 0.19 mL min^−1^, starting with 5% B and increasing linearly to 35% B in 45 min, followed by 15 min of re-equilibration at 5% B. The column was kept at 40 °C and detection was done with both a PDA detector (Waters) at 210–600 nm and an LTQ-Orbitrap FTMS hybrid mass spectrometer (Thermo Scientific, Bremen, Germany) in negative ionization mode. A mass resolution of 60,000 FWHM was employed for data acquisition. Eluting compounds were detected in full-scan mode in the *m*/*z* range of 90–1350. Separate LCMS/MS runs were performed by re-injecting a random set of extracts, using data-dependent acquisition in discovery mode by selecting the 3 most intense ions per full scan for fragmentation up to MS3. Some selected phenolic compounds were identified or quantified using authentic standards: procyanidin B1, B2 (Extrasynthese^®^, Genay Cedex, France) and B3 (APIN Chemicals Ltd.^®^, Compton, United Kingdom), catechin (APIN Chemicals Ltd.^®^), epicatechin (Sigma^®^, Zwijndrecht, the Netherlands), epicatechin-3-*O*-gallate (Extrasynthese^®^), quercetin-3-glucoside (Fluka^®^, Munich, Germany), piceid (APIN Chemicals Ltd.^®^) and resveratrol (Sigma^®^).

#### 3.2.2. GCMS Analysis

For the analysis of polar (primary) compounds, we used an untargeted GCMS platform. In view of limited sample amounts, we only analyzed the 24 exocarp samples from the mature stage. Extraction was according to the protocol described by Carreno-Quintero et al. [84]. Briefly, 10 mg of dry weight powder was extracted with 1.4 mL of methanol/water 75% (*v*/*v*) containing 8 µg mL^−1^ of ribitol (Sigma^®^) as the internal standard. After sonication and centrifugation, 500 µL of the supernatant was mixed with 375 µL of chloroform (−20 °C) and 750 µL of distilled water (4 °C). After a new centrifugation, aliquots (50 µL) of the upper (polar) phase were transferred to an insert placed in a 2 mL vial. All samples were dried overnight (16 h) by vacuum centrifugation (Savant^®^, SPD121P, Thermo Scientific) at room temperature and the vials were closed under an argon atmosphere using magnetic crimp caps. Prior to analysis, dried samples were derivatized online using a TriPlusRSH autosampling/injection robot (Thermo Scientific) [84,85]. First, 12.5 µL of o-methylhydroxylamine hydrochloride (20 mg mL^−1^ pyridine) was added to the samples and incubated for 30 min at 40 °C with agitation. Then, the samples were derivatized with 17.5 µL of *N*-methyl-*N*-trimethylsilyltrifluoroacetamide (MSTFA) for 60 min. An alkane mixture (C10-C32) was added to determine the retention indices of metabolites. The derivatized samples were analyzed by a GCMS system consisting of a Trace 1300 gas chromatograph (Thermo Scientific) with a PTV injector coupled to a TSQ8000 DUO-series triple quadrupole mass spectrometer (Thermo Scientific). One microliter of each sample was introduced to the injector at 70 °C using a split flow of 19 mL min^−1^. Chromatographic separation was performed using a VF-5ms capillary column (Varian, Palo Alto, CA, USA; 30 m × 0.25 mm × 0.25 mm) including a 10 m guardian column with helium as the carrier gas at a column flow rate of 1 mL min^−1^. The column effluent was ionized by electron impact at 70 eV. Mass spectra were acquired at a combined SRM and full scan mode with a *m*/*z* range of 50 to 600 at an ion source temperature of 290 °C. A solvent delay of 420 s was set.

#### 3.2.3. Untargeted Data Processing and Multivariate Statistical Analysis

Unbiased mass peak picking and alignment of the raw data sets from LCMS and GCMS were carried out separately for each tissue using MetAlign software [86]. Irreproducible individual mass signals (present in <3 samples) were filtered out using an in-house script called MetAlign Output Transformer (METOT) [87]. The remaining mass peaks, including molecular ions, in-source adducts (in case of LCMS), fragments and their natural isotopes, were subsequently clustered using MSClust software into so-called reconstructed metabolites (centrotypes) [88] according to their corresponding retention time and peak intensity pattern across samples. In the final LCMS dataset, the total number of non-detects, i.e., below the detection limit of 2500 ion counts per compound, was 12,154 and 14,308 for exocarp and seeds, respectively. These non-detects were subsequently filtered out when not present in all 3 or 4 biological replicates of at least one sample group. The values of the remaining non-detects (3394 and 2459 for exocarp and seeds, respectively) were randomized between 45% and 55% of the detection threshold, i.e., between 1125 and 1375. The resulting spreadsheets for exocarps (Supplemental File S1, Table S1) and seeds (Supplemental File S2, Table S1) with the relative intensity of each reconstructed metabolite in each sample were used for further statistical analyses. 

The on-line tool MetaboAnalyst was employed to compare the three developmental stages for each tissue [89]. The MSClust output was uploaded into this platform and was Log_10_-transformed and scaled by the Pareto method (mean-centered and divided by the square root of standard deviation of each variable). Principal component analysis (PCA) was used as an unsupervised approach. In addition, the heatmap plot was represented based on the 25 top-ranking metabolites according to Analysis of Variance (ANOVA) test. On this test, the *p*-values were adjusted using a false discovery rate (FDR) correction.

A multivariate statistical analysis was carried out using MATLAB software. An ANOVA simultaneous component analysis (ASCA), a common tool for analysis of metabolomics data [90], was applied to the Log-transformed data. The model comprised the following factors: HL vs. LL, K vs. NK, I vs. NI and their interactions. The significance of each factor was assessed by a permutation test using 1000 permutations, and Wilks Lambda as a test statistic [91]. Separate ASCA models were fitted to the data from each developmental stage to study the influence of the factors of interest on the overall metabolome of grape berry tissues. In addition to the ASCA analysis, the effect of the factors mentioned above on each metabolite was studied in more detail by N-way ANOVA. For each factor of interest, the *p*-values were adjusted for multiple comparisons using the Benjamini–Hochberg false discovery rate (FDR) approach. Adjusted *p*-values smaller than 0.05 were considered to be significant. For each factor, the significant metabolites showing the strongest effect (i.e., highest fold change (FC) values, estimated by the ANOVA) were considered for manual annotation.

The mass of the molecular ion was manually verified within the clustered mass signals of selected, reconstructed metabolites. Metabolites were then annotated using an in-house metabolite database based on comparisons of retention time, accurate mass and UV spectra, if available. On-line available metabolite databases (e.g., KNApSAcK) and literature on grape analyses were also employed for annotation. 

In the case of GCMS metabolites, the mass spectrum of each ion cluster was compared with that in available EI-spectral libraries, such as the NIST2014 and the Golm spectral database [92], as well as an in-house library of derivatized standards. In addition, the experimentally obtained RI was compared with reported RIs for verification of the automated spectra annotations. 

The level of annotation of compounds was performed following the rules described by Sumner et al. [93], being classified at four levels: identified metabolites by comparison with standards (level 1), putatively annotated compounds (level 2), putatively characterized compound classes (level 3), and unknown compounds (level 4).

### 3.3. Targeted Analysis

#### 3.3.1. Tocopherols

The extraction procedure for tocopherols was the same as recently described for chlorophylls and carotenoids [18]. Briefly, 20 mg DW of all 128 samples of the grape berry tissues (exocarp or seed) were extracted in 1.8 mL of chloroform/methanol (1:1) with both 0.1% (*w*/*v*) butylated hydroxytoluene (BHT) as an antioxidant and Sudan 1 (0.5 µg mL^−1^, Sigma^®^) as the internal standard (IS). The samples were vortexed, sonicated and centrifuged. The supernatant was dried for 1 h in a Speedvac and prior to analysis, the dried samples were dissolved in 200 µL ethylacetate containing 0.1% (*w*/*v*) BHT, again sonicated and centrifuged, and the final supernatant (180 µL) was transferred to amber-colored 2 mL HPLC vials. Samples (20 μL for injection) were analyzed using an HPLC (Waters Alliance e2695 Separations Module, Milford, MA, USA) coupled to a fluorescence detector (Waters 2475) with excitation at 296 and emission at 340 nm. Separation was performed on a reverse-phase C30 column (250 × 4.6 mm i.d., S-5 μm—YMC Carotenoid, Komatsu, Japan) kept at 35 °C with a flow rate of 1 mL min^−1^. The three tocopherol species detected (i.e., α-, γ- and δ-tocopherol) were identified based on comparisons of retention times with authentic standards. Waters Empower 3 software (Waters, Milford, MA, USA) was used for data processing. The total tocopherol content (µg per g of DW tissue) was obtained by adding up the levels of the three detected tocopherols.

#### 3.3.2. Sugars

All 24 exocarp samples from the mature stage (10 mg DW) were extracted with methanol/water 75% (*v*/*v*). After sonification (10 min), followed by centrifugation (10 min) at maximal speed (16,100× *g*), the supernatant was transferred to new Eppendorf tubes and stored at −20 °C until use. For the sugar analysis, 20 µL was transferred to plastic vial and dried in a vacuum centrifugation without heating. The residue was resuspended in 0.2 mL MiliQ water and vortexed thoroughly. The residue dissolved in water was injected into a Dionex HPLC system (ICS 5000+DC) to analyze the sugar content, using a CarboPac PA 1, 4 × 250 mm column preceded by a guard column (CarboPac PA 1, 4 × 50 mm). Mono-, di-, and tri-saccharides were separated by elution in an increasing concentration of NaOH (20–350 mM) with a flow rate of 1 mL min^−1^. Peaks were identified by co-elution of standards. The sugar amount was expressed in mg of sugar per g of dry material.

#### 3.3.3. Total Soluble Phenolics

The Folin–Ciocalteu colorimetric method was used for total phenolics quantification in all 24 exocarp and 24 seed samples from the mature stage [94]. Ten mg DW were extracted in 300 μL of 75% (*v*/*v*) methanol/water with 0.1% formic acid (FA), and after vortexing (10 s) and sonication (15 min), samples were centrifuged (16,100× *g*) for 15 min and the supernatant was collected. After that, 50 μL of extract was added to 300 μL of 10% (*v*/*v*) Folin reagent and incubated for 5 min in the dark before adding 300 μL of 6% (*w*/*v*) sodium carbonate. After 2 h of incubation in the dark, the absorbance was measured at 765 nm. The phenolic contents were determined using a gallic acid (Sigma^®^) calibration curve and expressed as mg of gallic acid equivalents [GAE]/g DW tissue.

#### 3.3.4. Lipid Peroxidation Products

The 16 green exocarp and 12 mature seed samples from the two distinct light microclimates were selected for the analysis of lipid peroxidation products. Ten mg DW were extracted in 800 µL of 0.5% (*w*/*v*) 2-thiobarbituric acid (TBA) freshly dissolved in 20% (*v*/*v*) trichloroacetic acid (TCA), and 800 µL of water was added. The mixture was vortexed, heated at 95 °C for 30 min in a water bath, cooled on ice and centrifuged at max speed for 10 min. The absorbance of the supernatant was measured at both 532 and 600 nm. Lipid peroxidation product levels were calculated as described by Hodges et al. [95], and expressed in thiobarbituric acid-reactive-substances (TBARS) per g DW using malondialdehyde (MDA) as a standard. 

#### 3.3.5. Statistical Analysis

Analysis of Variance tests (two-way ANOVA) were applied, followed by post hoc multiple comparisons using the Bonferroni test whenever the factors had significant effects (GraphPad Prism version 5.00 for Windows, GraphPad Software, La Jolla, CA, USA).

## 4. Conclusions

We previously showed that both the exocarp and seed of berries from the white grape variety Alvarinho are photosynthetically active and more so in berries exposed to full sunlight (HL microclimate) than in those of shaded locations in the vines (LL microclimate), especially at the green stage of their development [18]. Here, we used unbiased, comprehensive LCMS- and GCMS-based metabolomics approaches, as well as targeted analyses of selected key compounds for grape/wine quality traits, in order to get more insight into the effects of these contrasting canopy light microclimates, as well as into the effects of soil irrigation and foliar kaolin spraying, on the metabolome composition of berry exocarps and seeds. Both strategies are regularly applied in viticulture as potential mitigation against abiotic stress. Our results indicate the significant influence of the microclimate in both photosynthetically active berry tissues, suggesting a potential role for in situ berry photosynthesis in contributing carbon-skeletons and energy for the biosynthesis of berry components during development and ripening. More experimental research, e.g., by specifically applying artificial shading and exposing grapes to additional light of specific wavelengths, is needed to get a better understanding of the exact role of berry photosynthesis in the final grape quality. In addition, the foliar kaolin application and especially the irrigation treatment appeared to exert their own or combined additional modulating effects on the metabolome of these two berry tissues, possibly due to their direct or indirect influence on photosynthesis in both the leaves and berries. Several compounds affected by these mitigation treatments are also relevant to viticulture, e.g., modulation of a series of phenolic compounds including mono- and polymers of flavan-3-ols, suggesting that good management of these treatments by farmers is necessary and may even further optimize their products by fine-tuning the berry metabolome. Moreover, it is also important to emphasize that this was an exploratory study which aimed to contribute new knowledge and generate hypotheses for future experiments. In addition, for more robust conclusions, more biological replicates and repeating campaigns are helpful in order to possibly link the grape berry metabolite composition to year-to-year variations in wine quality.

## Figures and Tables

**Table 1:** Levels of significance (*p* values) obtained by ANOVA simultaneous component analysis (ASCA), for the effects of the various growth conditions and their interactions on the exocarp and seed metabolite composition, based on either the LCMS or GCMS analysis, during berry ripening (G—Green, V—*Véraison*, M—Mature). Significant effects (*p* ≤ 0.05) are highlighted by the grey color; interactions that appear insignificant were omitted. The numbers between brackets indicate the total number of significant (FDR-adjusted *p* ≤ 0.05) metabolites from N-way ANOVA.

	LCMS Data						GCMS Data
	Exocarp			Seed			Exocarp
Growth Conditions	G	V	M	G	V	M	M
Soil irrigation	-	0.001 (78)	0.002 (48)	-	0.016 (30)	0.264	0.147
Kaolin	0.472	0.112	0.165	0.262	0.197	0.145	0.036 (0)
Berry microclimate (HL/LL)	0.001 (95)	0.001 (88)	0.001 (154)	0.012 (26)	0.115	0.006 (31)	0.003 (3)
Irrigation × Kaolin	-	0.044 (0)	0.043 (0)	-	0.084	0.194	0.001 (10)

## Data Availability

The data presented in this study are available in Supplementary Materials.

## Supplementary Materials

The following are available online at https://www.mdpi.com/article/10.3390/metabo11040205/s1, Supplemental File S1–LCMS and GCMS data and statistical results for exocarps (Tables S1–S9), Supplemental File S2–LCMS data and statistical results for seeds (Tables S1–S5), Figure S1. LCMS chromatograms of exocarp, Figure S2. LCMS chromatograms of seeds, Figure S3. Heatmaps with the most significant metabolites affected by developmental stages in exocarp (a) and seed (b), Figure S4. Principal component analysis (PCA) score plots of LCMS metabolite data for exocarp and seed at *véraison* stage, Figure S5. Example profile of polar metabolites obtained by GCMS analysis in mature exocarp, Figure S6. PCA score plots of GCMS metabolite data for mature exocarp, Figure S7. Relative abundance of some selected primary metabolites in the exocarp of mature berries as detected by GCMS, Figure S8. Sum of relative intensities of the six main flavonol peaks as detected by LCMS for the exocarp, Figure S9. Sum of relative intensities of the two viniferins as detected by LCMS for seeds, Figure S10. Relative abundance values for some selected metabolites detected by LCMS in exocarps and seeds under non-irrigation and irrigation conditions. Figure S11, α-tocopherol contents in exocarps (a, c and e) and seeds (b, d and f), Figure S12. γ-tocopherol contents in exocarps and seeds, Figure S13. δ-tocopherol contents in exocarps and seeds.
