# Supplementary material for: Metabolomics of Photosynthetically Active Tissues in White Grapes: Effects of Light Microclimate and Stress Mitigation Strategies
Source: Metabolites. 2021 Mar 30;11(4):205. doi: 10.3390/metabo11040205 (PMC8067353; doi:10.3390/metabo11040205)
Supplement: Supplementary file 1 [file metabolites-11-00205-s001.zip › Supplementary Materials_vs13.docx]

Metabolomics of Photosynthetically Active Tissues in White Grapes: Effects of Light Microclimate and Stress Mitigation Strategies

Andreia Garrido ^1,^*, Jasper Engel ^2,3^, Roland Mumm ^2^, Artur Conde ^1,4^, Ana Cunha ^1,4,5,^*^,†^ and Ric C. H. De Vos ^2,†^

^1^ Centre for the Research and Technology of Agro-Environmental and Biological Sciences (CITAB),
University of Minho, Campus de Gualtar, 4710-057 Braga, Portugal; arturconde@bio.uminho.pt

^2^ Business Unit Bioscience, Wageningen Plant Research, Wageningen University and Research (Wageningen-UR), PO Box 16, 6700 AA Wageningen, The Netherlands; jasper.engel@wur.nl (J.E.);
roland.mumm@wur.nl (R.M.); ric.devos@wur.nl (R.C.H.D.V.)

^3^ Business Unit Biometris, Wageningen Plant Research, Wageningen University and Research (Wageningen-UR), PO Box 16, 6700 AA Wageningen, The Netherlands

^4^ Centre of Molecular and Environmental Biology (CBMA), University of Minho, Campus de Gualtar,
4710-057 Braga, Portugal

^5^ Centre of Biological Engineering (CEB), University of Minho, Campus de Gualtar, 4710-057 Braga, Portugal

***** Correspondence: andreiagarrido@sapo.pt (A.G.); accunha@bio.uminho.pt (A.C.)

† Equal senior authorship.

**Supplementary Materials**

**(a)** Green

**(b)** *Véraison*

**(c)** Mature

**Figure S1.** Liquid chromatography mass spectrometry (LCMS) chromatograms of exocarp at different berry developmental stages: green (**a**), *véraison* (**b**) and mature (**c**). Numbers above peaks represent, from top to bottom, the retention time and mass signals, respectively. The three chromatograms are in the same scale. Annotations of compounds, if known, are provided in Supplemental File 1, Table S1.

**(a)** Green

**(b)** *Véraison*

**(c)** Mature

**Figure S2.** LCMS chromatograms of seeds at different berry developmental stages: green (**a**), *véraison* (**b**) and mature (**c**). Numbers above peaks represent, from top to bottom, the retention time and mass signals, respectively. The three chromatograms are in the same scale. Annotations of compounds, if known, are provided in Supplemental File 2, Table S1.


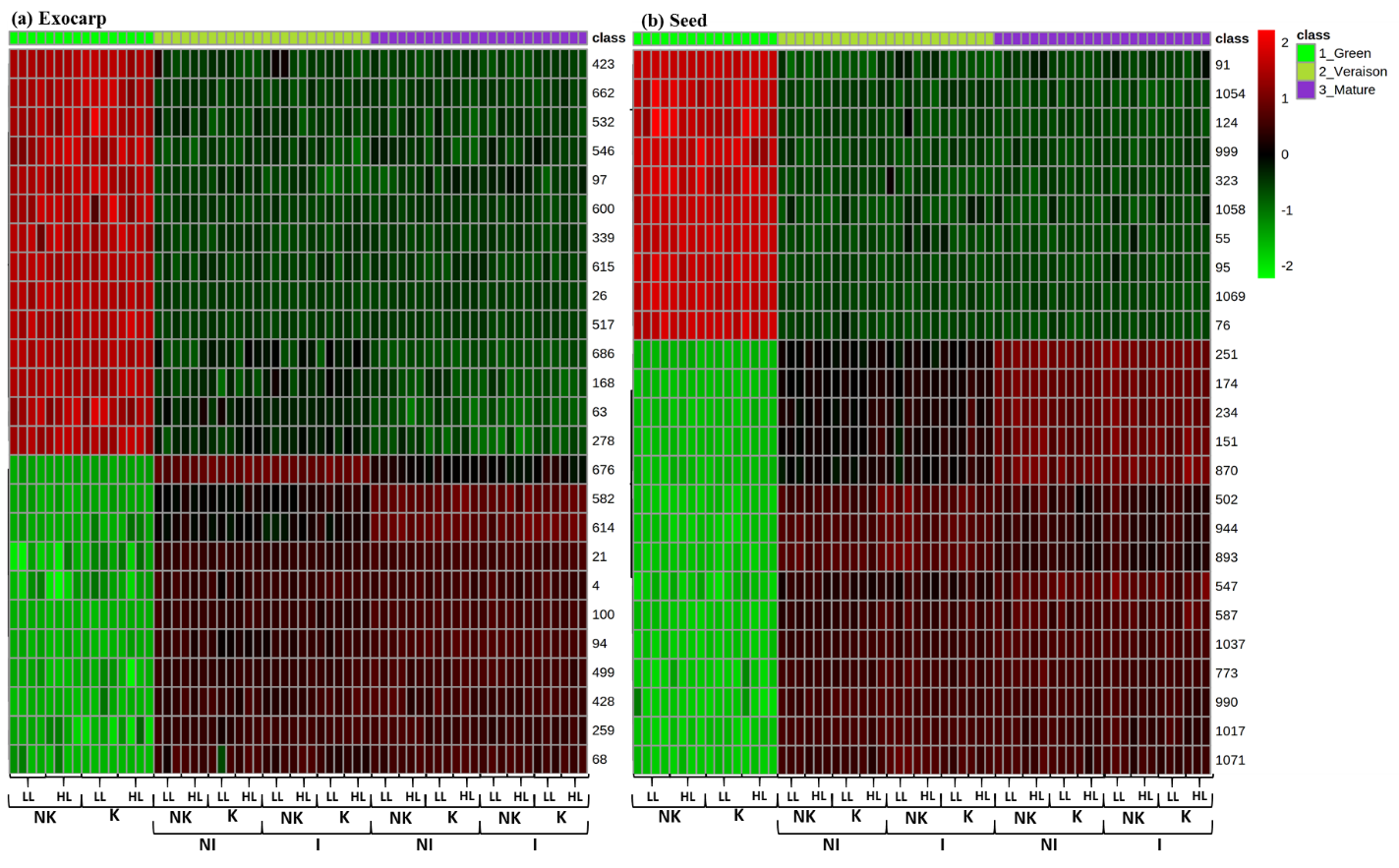
**Figure S3.** Heatmaps with the most significant LCMS-metabolites affected by developmental stages in exocarp (**a**) and seed (**b**). Heatmap plot with the 25 top-ranking metabolites from Analysis of Variance (ANOVA). Metabolites are represented as numbers on the right-hand side of the plot, using the metabolite number (metabolite ID) obtained from the data processing. Developmental stages are represented from left to right side of the plot by order, as well as, the replicates for low and high light (LL and HL, respectively) grape berries grown under the four combinations of the two treatments applied: irrigation (I)/ non-irrigation (NI) x kaolin (K)/non-kaolin (NK).


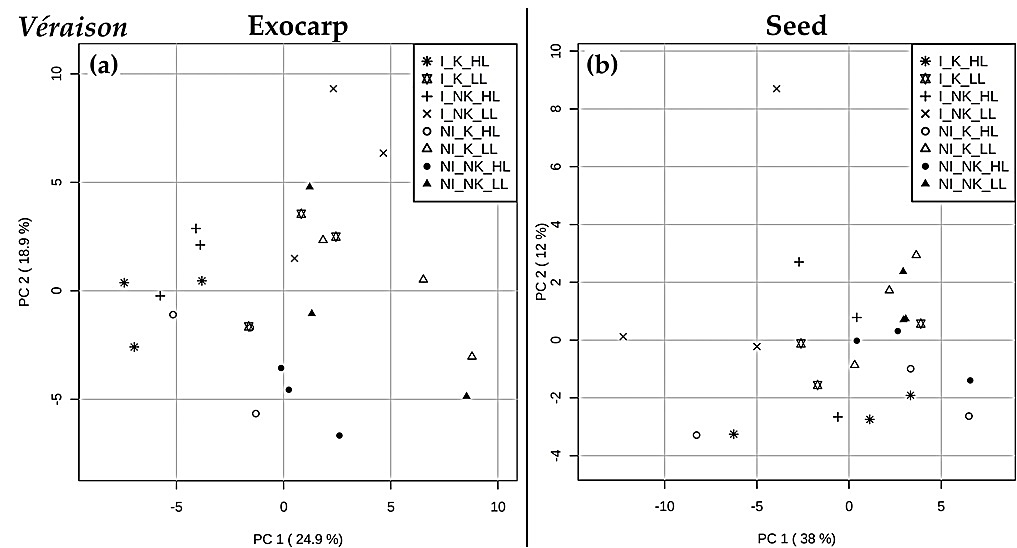


**Figure S4.** Principal component analysis (PCA) score plots of the liquid chromatography mass spectrometry (LCMS) metabolite data for exocarp and seed at *véraison* stage, including all microclimates and treatments (*n* = 3).


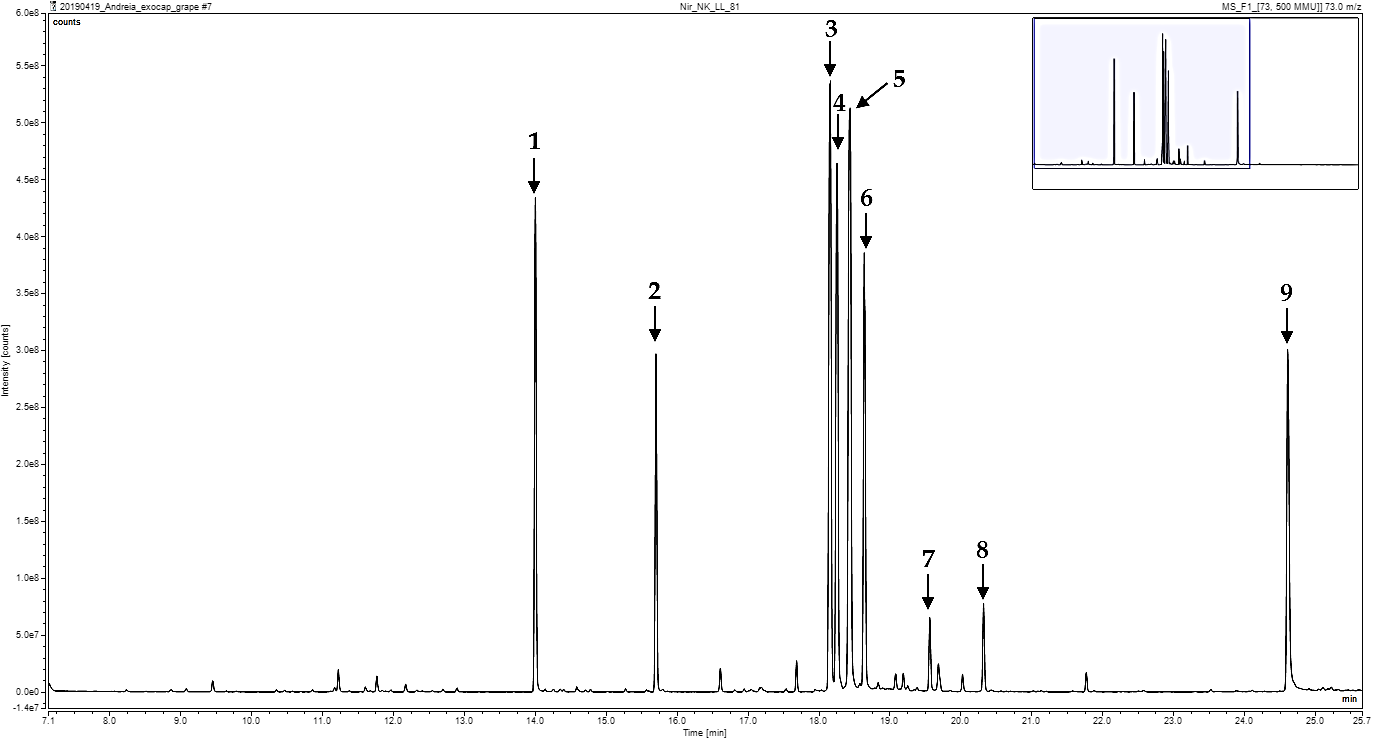


**Figure S5.** Gas chromatography mass spectrometry (GCMS) raw chromatogram of polar metabolites extracted from the exocarp of mature berries. **1 -** Malic acid (ID 1851); **2 -** Tartaric acid (ID 3244); **3 -** Fructose, isomer 1 (ID 5298); **4 -** Fructose, isomer 2 (ID 5745); **5 -** Glucose, isomer 1 (ID 6481); **6** - Galactose, isomer 2 (ID 7204); **7 -** D-Gluconic acid (ID 8359); **8 -** Myo-inositol (ID 9692); **9 -** Sucrose (ID 11573).


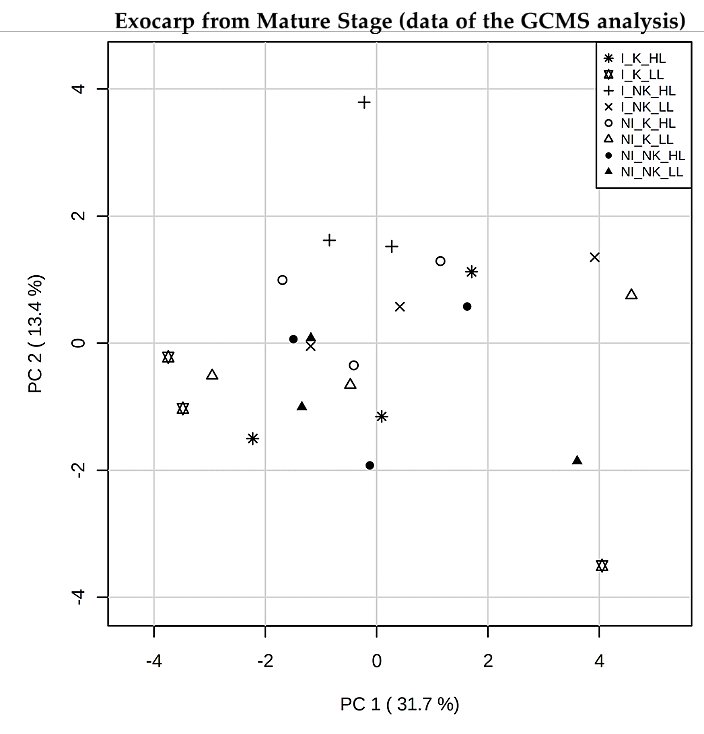


**Figure S6.** Principal component analysis (PCA) score plots of the gas chromatography mass spectrometry (GCMS) metabolite data for exocarp at mature stage, including all microclimates and treatments (*n* = 3). The abbreviations in the legend represent: NI - Non-Irrigation; I - Irrigation; NK - Non-kaolin; K - Kaolin; LL - Low Light microclimate; HL - Hight Light microclimate.

**Figure S7.** Relative abundance of some selected primary metabolites in the exocarp of mature berries as detected by GCMS (means and SD of *n* = 6, data of high light and low light microclimate combined): (**a**) L-Alanine (ID 186); (**b**) Quininic acid (ID 4990); (**c**) Fructose, isomer 1 (ID 5298); (**d**) Fructose, isomer 2 (ID 5745); (**e**) Glucose, isomer 1 (ID 6481); (**f**) Galactose, isomer 2 (ID 7204); (**g**, **h**) Unidentified sugar (ID 7816 and 9117); (**i**) Sucrose (ID 11573). Berries grown under the four combinations of the two treatments applied: irrigation (I)/ non-irrigation (NI) x kaolin (K)/ non-kaolin (NK). Statistical analysis (one-way ANOVA, *p* ≤ 0.05) was applied after data Log_2_ transformation. Statistical notation above the bars: letters refer to differences between treatment combinations.

**Figure S8.** Flavonols in the exocarp. (**a**) Sum of relative intensities of the six main flavonol peaks as detected by LCMS (means and SD of *n* = 8-12) at two canopy microclimates (LL and HL; independent of mitigation treatment) and at three developmental stages (green, *véraison* and mature). The flavonols considered are: rutin (quercetin-3-*O*-rutinoside) (ID 462), quercetin 3-*O*-glucoside (ID 492), quercetin 3-*O*-glucuronide (ID 505), kaempferol-3-glucoside (ID 545), kaempferol glucuronide (ID 588) and isorhamnetin hexoside (ID 583) (Supplemental File 1, Table S5-S7). Statistical analysis (two-way ANOVA, *p* ≤ 0.05) was applied after data Log_2_ transformation. (**b**) Quercetin 3-*O*-glucoside amount (mg/g of dry weight (DW)) in exocarp tissue grown at two canopy microclimates and from three developmental stages. Statistical notation above the bars: the capital letters refer to differences between developmental stages for the same microclimate, while the lowercase letters refer to differences between the two light microclimates for each stage.

**Figure S9.** Sum of relative intensities of the two viniferins as detected by LCMS (means and SD of *n* = 8-12) for seed tissue at two canopy microclimates (LL and HL; independent of mitigation treatment) and at three developmental stages (green, *véraison* and mature). The viniferins considered are: (+)-alpha-viniferin-hexoside (ID 990) and viniferin 3''-glucoside (ID 1027). Statistical analysis (two-way ANOVA, *p* ≤ 0.05) was applied after data Log_2_ transformation. Statistical information is the same as in Figure S7.

**Figure S10.** Relative abundance of some selected metabolites detected by LCMS (*n* = 8-12, +SD) in exocarps and seeds under non-irrigation (grey bars) and irrigation conditions (blue bars) applied at two berry developmental stages (*véraison* and mature) (data of HL and LL berries combined). For exocarp: (**a**) D-Fructose 1,6-bisphosphate (ID 79), (**b**) arginine (ID 77). For seed: (**c**) hexose sugar (ID 21) and (**d**) coutaric acid (ID 315). Statistical analysis (two-way ANOVA, *p* ≤ 0.05). Statistical notation above the bars: the capital letters refer to differences between developmental stages for the same treatment condition, while the lowercase letters refer to differences between the control and irrigation treatment, within each developmental stage.

**Figure S11.** α-tocopherol contents (mean values of *n* = 3 or 4 biological replicates, +SD) in exocarps (**a**, **c** and **e**) and seeds (**b**, **d** and **f**) of berries from grape clusters, grown either low light (LL) or high light (HL) microclimate conditions in the canopy. Kaolin (K) or no kaolin (NK) was applied to the plant leaves before fruit set; after green berries were developed, plants were either irrigated (I) or non-irrigated (NI). Samples were collected at three development stages: green (**a** and **b**), *véraison* (**c** and **d**) and mature (**e** and **f**). Statistical notations above the bars: at each developmental stage, the capital letters refer to differences between the two light microclimates within the same treatment, while the lowercase letters refer to differences between treatment combinations within the same light microclimate (values with a common letter or no letter at all, indicate no significant differences; two-way ANOVA, *p* ≤ 0.05). Notation with an asterisk means that only one factor (in this case the irrigation) was significant.

**Figure S12.** γ-tocopherol contents (mean values of *n* = 3-4 biological replicates, +SD) in exocarps (**a**, **c** and **e**) and seeds (**b**, **d** and **f**). All the microclimate conditions, treatment combinations, and statistical information are the same as in Figure S10.

**Figure S13.** δ-tocopherol contents (mean values of *n* = 3-4 biological replicates, +SD) in exocarps (**a**, **c** and **e**) and seeds (**b**, **d** and **f**). All the microclimate conditions, treatment combinations, and statistical information are the same as in Figure S10.
